# The association between sedentary behaviour and risk of anxiety: a systematic review

**DOI:** 10.1186/s12889-015-1843-x

**Published:** 2015-06-19

**Authors:** Megan Teychenne, Sarah A Costigan, Kate Parker

**Affiliations:** 1 grid.1021.20000000105267079Centre for Physical Activity and Nutrition Research, School of Exercise, and Nutrition Sciences, Deakin University, 221 Burwood Hwy, Burwood, VIC 3125 Australia; 2 grid.266842.c000000008831109XPriority Research Centre for Physical Activity and Nutrition, University of Newcastle, Callaghan, NSW Australia

**Keywords:** Sitting time, Television viewing, Mental health, Anxiety

## Abstract

**Background:**

Previous research has linked sedentary behaviour (SB) to adverse physical health outcomes in adults and youth. Although evidence for the relationship between SB and mental health outcomes (e.g., depression) is emerging, little is known regarding risk of anxiety.

**Methods:**

A systematic search for original research investigating the association between SB and risk of anxiety was performed using numerous electronic databases. A total of nine observational studies (seven cross-sectional and two longitudinal) were identified. Methodological quality of studies was assessed and a best-evidence synthesis was conducted.

**Results:**

One cross-sectional study demonstrated a strong methodological quality, five cross-sectional studies demonstrated a moderate methodological quality and three studies (two cross-sectional one longitudinal) received a weak methodological quality rating. Overall, there was moderate evidence for a positive relationship between total SB and anxiety risk as well as for a positive relationship between sitting time and anxiety risk. There was inconsistent evidence for the relationship between screen time, television viewing time, computer use, and anxiety risk.

**Conclusion:**

Limited evidence is available on the association between SB and risk of anxiety. However, our findings suggest a positive association (i.e. anxiety risk increases as SB time increases) may exist (particularly between sitting time and risk of anxiety). Further high-quality longitudinal/interventional research is needed to confirm findings and determine the direction of these relationships.

**Electronic supplementary material:**

The online version of this article (doi:10.1186/s12889-015-1843-x) contains supplementary material, which is available to authorized users.

## Background

Sedentary behaviour (i.e. activities which require minimal body movement resulting in low energy expenditure similar to resting level [1.0 to 1.5 metabolic equivalent (METs)] [1]) includes behaviours such as sitting for a range of purposes (e.g. work, travel), and screen-based activities such as computer use, electronic gaming, and television viewing. Time spent in sedentary behaviour has emerged as a potentially important indicator of health in adult populations [2], independent of achieving sufficient physical activity. Among the general adult population some evidence suggests sedentary behaviour is associated with increased risk of developing various chronic diseases (e.g. overweight and obesity [3], cardiovascular disease [4], osteoporosis [5], type two diabetes [3], and various cancers [6]). However, reviews of prospective studies suggest that there is yet insufficient or no evidence to conclude relationships between sedentary behaviour and certain health outcomes (e.g. adulthood weight gain, cardiovascular disease risk, and some cancers) [7, 8]. A range of health consequences of sedentary behaviour in children and adolescents have also been identified (e.g. unfavourable body composition, decreased fitness, lowered scores for self-esteem, and pro-social behaviour and decreased academic achievement [9], sleep problems, musculoskeletal pain, depression, and poor psychological well-being [10]). However, like that of the adult literature, reviews of prospective studies in young people suggest that there still remains insufficient evidence to conclude associations between sedentary behaviour and some health indicators (e.g. body weight, blood pressure, bone mass) [11].

The association between sedentary behaviour and mental health issues such as depression [12, 13] and self-esteem [14] have been explored within some population groups (e.g., women, adolescents), and on the balance it has been suggested that a positive relationship exists between most sedentary behaviours and depression [12] and self-esteem [9]. However little is known regarding the relationship between sedentary behaviour and other mental illnesses such as anxiety.

Anxiety is a mental illness that affects approximately 14 % of Australians adults [15] and 15 % of 16–24 year olds [16], with global estimates indicating anxiety to affect over 27 million people [17]. Anxiety is characterised by excessive and persistent (yet often unrealistic) worry which can inhibit one’s ability to carry out activities of daily living [18]. Physiological symptoms can include: pounding heart; difficulty breathing; upset stomach; muscle tension; sweating; and, feeling faint, or shaky [18]. The illness has been shown to be linked to other serious diseases such as increased risk of cardiovascular disease and cancer [19] with estimates that anxiety and depression together contribute to 10 % of the total burden of disease in Australian women [20]. Considering anxiety has such a large impact on society (e.g. healthcare costs, quality of life, and life expectancy) and is highly prevalent across the life span, it is important to understand the behavioural factors that may be linked to it.

It has been hypothesised that sedentary behaviour may lead to anxiety through biological pathways. For example, engaging in screen-based entertainment, such as video gaming, has been shown to increase the arousal of the central nervous system (CNS) [21], which could potentially lead to increased levels of anxiety. Additionally, screen-based sedentary behaviours have been linked to disrupted sleeping patterns which may also cause elevated levels of anxiety [22]. Given the plausibility of these short-term effects of sedentary behaviours on mood (i.e. distraction, CNS arousal, sleep), it is likely that the cumulative effects of these behaviours may further result in longer-term impacts on anxiety risk. Alternatively, the link between sedentary behaviour and anxiety could be explained by poor metabolic health. For example, research has shown sedentary behaviour to be linked to an increased risk of type 2 diabetes [3], a disease which is subsequently associated with poor mental health [23]. On the other hand, it could be hypothesised that those with mental illness (e.g. anxiety) tend to engage in sedentary behaviour more than those without the illness.

Although research has indicated an inverse association between physical activity and anxiety may exist [24–26], currently the evidence regarding the association between sedentary behaviour and anxiety is unclear. Thus, the aim of this systematic review is to investigate the association between sedentary behaviour and risk of anxiety across the lifespan.

## Methods

### Search strategy

A structured electronic search (employing PRISMA reporting guidelines) of publication years from 1990 (through November 2014) was conducted, since the 1990’s saw an increase in sedentary behaviour levels of the population with the widespread use of online technology [27]. Databases included: CINAHL complete, Medline/Medline complete, PsychARTICLES, Psychology, and Behavioral Sciences Collection, PsychINFO, and SPORTDiscus. The following search strings were used: (anxiety OR anxious OR mental health OR mental illness) AND (sedentary behav* OR sitting OR TV OR television OR computer OR screen). These strings were further limited to peer-reviewed publications written in English. First, title, and abstracts of articles identified in the search process were assessed for suitability. Second, full-text articles were retrieved, and assessed for inclusion. Third, reference lists from retrieved full-text articles were searched. Additional records were identified through other sources (i.e. Authors own bibliographic library).

### Study selection criteria

For the purpose of this review, risk of anxiety was defined as either the likelihood of developing or experiencing an anxiety disorder or non-clinical anxiety symptoms. Studies were considered eligible if they: (1) examined ‘healthy’ children, adolescents, or adults (i.e. those who were not patients suffering from underlying chronic physical conditions that may confound results); (2) examined risk of anxiety specifically; (3) assessed screen-based sedentary behaviour or sitting time; (4) and involved a cross-sectional, longitudinal, or experimental study design. However, only intervention studies which primarily show the relation between sitting and anxiety (i.e. not just the effect of an intervention on anxiety) were eligible to be included. Conference abstracts, dissertations, theses, and articles published in non-peer-reviewed journals were not included for review.

### Data extraction

Key study characteristics of the identified studies were extracted including: the country of origin, size/source of study population, study design, domain (e.g. leisure time sitting, occupational sitting, total sitting), measures used, indicator of sedentary behaviour (e.g. computer use, television viewing, screen time, sitting), and study results in terms of the association between sedentary behaviour and risk of anxiety.

### Methodological quality

A modified version of an eight-component rating scale [28] was used to determine methodological quality of each study. The tool assesses eight methodological components of research studies including: selection bias (e.g. response rate, representativeness), study design (e.g. cohort, RCT, etc.), confounders (e.g. controlling for confounders such as age, socio-economic position etc.), blinding (e.g. awareness of group allocation, etc.), data collection methods (e.g. valid, reliable), withdrawals, and dropouts (e.g. percent providing full data), intervention integrity (e.g. percent receiving intervention) and analyses (e.g. appropriateness of study design). In regards to the observational studies that were assessed, the tool was modified so that those studies were not scored on 1) the blinding component or 2) other intervention-specific criteria within any other components. Thus, since only observational studies were included in this review, a maximum number of six components were scored. Each of the components was given an overall quality score of weak, moderate, or strong. If a component was not described, authors of those studies were attempted to be contacted to provide this information. In the instance that authors of those studies did not respond, the (undescribed) component was given a weak rating. Once each component was rated, an overall study rating of weak (if ≥2 of the components were scored weak), moderate (if <3 components were scored strong with no more than one weak score), or strong (if ≥3 components were scored strong) was given to each study. Two reviewers (SAC and KP) independently assessed the methodological quality of studies meeting the inclusion criteria. Scoring discrepancies were resolved via consensus and inter-rater reliability was calculated using percentage agreement.

### Best-evidence synthesis

In order to draw conclusions on the association between sedentary behaviour and anxiety risk with regards to the methodological quality of studies, a best-evidence synthesis was conducted, which was based on previous systematic reviews in the area of sedentary behaviour and health outcomes [7, 11]. As such, three levels of evidence considered:

1. Strong evidence: defined as consistent (i.e. at least 75 % of studies show results in same direction) results in ≥2 high quality studies
2. Moderate evidence: defined as consistent results in one high quality study and at least one weak quality study; or consistent results in ≥2 weak quality studies
3. Insufficient evidence: defined as having only one available study; or inconsistent results in ≥2 studies.

## Results

**Fig. 1:**
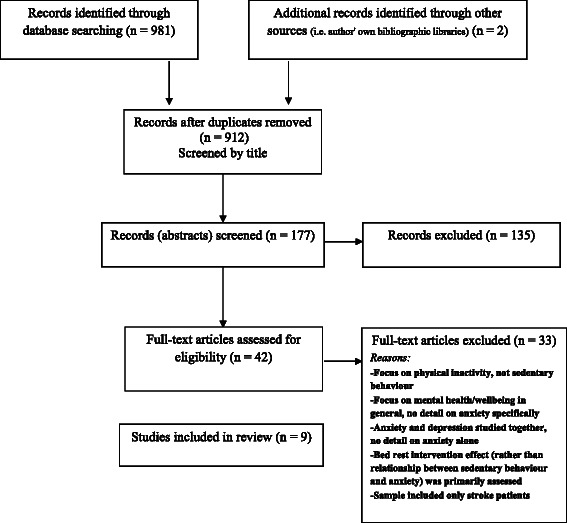
Flow of study selection through the phases of the review

Literature searching yielded 983 studies (see Fig. 1). A total of 71 duplicates were removed and thus 912 studies were screened by title. After further screening of abstracts (n = 177) and full papers (n = 42) a total of nine studies were included in the review (see Table 1). Most studies employed a cross-sectional study design (7/9) and two a longitudinal design. Samples sizes ranged from 189 [29] to 13,470 [30]. Descriptive characteristics of the nine studies are outlined in Table 1. A total of two studies included children/adolescents [30, 31], seven included adults [29, 32–36] and one longitudinal study examined adolescents with follow-up conducted during adulthood [37]. Most studies (7/9) measured sedentary behaviour using self-report methods such as the Global Physical Activity Questionnaire [35] or a modified version of the International Physical Activity Questionnaire [33]. Of those studies that utilised self-report measures, two measured screen-based entertainment (i.e. television/computer use/electronic game use) [31, 34], two examined sitting (either occupational sitting [33] or overall sitting [35]), one examined television viewing [29], one compared television viewing, computer use, and overall screen time [32], whilst one study compared six different forms of sedentary behaviour (total sitting, sitting at the computer, sitting watching television, transport-related sitting, work-related sitting, leisure-time sitting) [36]. Only one study utilised objective measures of sedentary behaviour (i.e. overall sitting time) using accelerometers [37], and one study used parent (proxy) report of screen-based entertainment [30]. Risk of anxiety (i.e. anxiety symptoms) was measured using various self-report measures including the Screen for Child Anxiety Related Emotional Disorders (SCARED) [31], the Behavioral Risk Factor Surveillance Survey [29], the Kessler Psychological Distress Scale (K10) [33], the General Health Questionnaire (GHQ-12) [35], the depression, anxiety and stress scale [36] and the Achievement Motivation Test (AMT) [37]. Interview methods were used in one study which utilised the Composite International Diagnostic Interview (CIDI) to determine presence of anxiety disorder [32], whilst a parent (proxy) report measure of the Strengths and Difficulties Questionnaire (SDI) (which specifically examined the Emotional Symptoms subscale [EM], an indicator of anxiety) was utilised in one other study [30] (Table 1).

**Table 1:** Studies investigating the association between sedentary behaviour and anxiety risk

Paper	Study details	Domain	Anxiety indicator	Sedentary behaviour indicator	Association	Methodological quality score
Cao et al. (2011) China [31]	Cross sectional	Leisure time	The 41-item Screen	Self report open ended question regarding TV & computer use (hrs per day)	+	Strong
			for Child Anxiety Related Emotional Disorders (SCARED)			
	5003 junior high school students (11–16 years)					
de Wit et al. (2011) The Netherlands [32]	Cross sectional	Leisure time	Composite International Diagnostic Interview (CIDI, WHO version 2.1)	Self report TV & computer use in leisure time	Total screen = +	Weak
	2353 (1701 with current diagnosis, 652 controls) adults aged 18-65					
					TV = +	
					Computer = 0	
Granner et al. (2010) USA [29]	Cross sectional	Total daily - including occupational, leisure time, and travel	Question from Behavioral Risk Factor Surveillance Survey asking “number of days last month felt worried, tense, anxious”	Self report TV, and sitting away from home, at work, drive, and at work (data from Nurses Health Study)	TV = +	Weak
	189 African American and Caucasian adult women aged 18–60 yrs					
					Total sitting = 0	
Griffiths et al. (2010) UK [30]	Cross sectional	Leisure time	Strengths and Difficulties Questionnaire (SDQ)	Proxy report of TV, computer use, and electronic games	Girls = −	Moderate
	13,470 5 yr old children				Boys = 0	
Kilpatrick et al. (2013) Australia [33]	Cross sectional	Occupational	Kessler Psychological Distress Scale (K10)	Self report modified from IPAQ for occupational sitting time	+	Moderate
	3367 state government adult employees (mean age 46.2 yrs)					
Rebar et al. (2014) Australia [36]	Cross sectional	Total daily – including occupational, leisure time, and travel	21-item depression, anxiety, and stress scale	Self report 10-item Workforce Sitting	Total SB = +	Moderate
					Computer = +	
	1104 Australian adults (mean age 58 years)					
				Questionnaire	Transport SB = +	
					Work sitting = 0	
					Leisure sitting = 0TV = 0	
Sanchez-Villegas et al. (2008) Spain [34]	Longitudinal	Leisure time	Participants were asked: Have	Self report sedentary index (TV & computer use)	0	Moderate
	10,381 adult University graduates					
			you ever been diagnosed of anxiety by a health			
	(7991 with data on SB)					
			professional?			
Sloan et al. (2013) Singapore [35]	Cross sectional	Total daily - including occupational, leisure time, and travel	General Health Questionnaire-12 (GHQ-12)	Self report global physical activity questionnaire (daily sitting time)	+	Moderate
	4337 Singapore citizens aged 18–79 yrs					
Uijtdewilligen et al. (2011) The Netherlands [37]	Longitudinal	Total daily - including all domains	Achievement Motivation Test (AMT) assessed achievement motivation, facilitating anxiety, debilitating anxiety, and social desirability	Objective data using accelerometers (total sedentary time)	+	Weak
	217 participants followed from adolescence to adulthood (age 13 to 42 years)					

## Methodological quality

Methodological quality scores are provided in Table 1. Initial agreement between reviewers was 90 % (K = 0.82) on the items. Overall, one cross-sectional study [31] demonstrated a strong methodological quality, five cross-sectional studies [30, 33–36] demonstrated a moderate methodological quality and three studies (two cross-sectional [29, 32], one longitudinal [37]) received a weak methodological quality rating. All of the studies were missing essential information regarding the methodological quality. For example, only three studies included reliable and valid measures for both sedentary behaviour and anxiety [33, 36, 37], and only two studies demonstrated a high retention rate (i.e. 80-100 %) [31, 34]. Further detail of the scoring of methodological quality of each study is provided in (see Additional file 1: Table S2).

### Main findings

Of the 9 studies included in this review, five (four cross-sectional [29, 31, 33, 35], one longitudinal [37]) found a positive association between sedentary behaviour and risk of anxiety (i.e. increased sedentary behaviour was linked to increased risk of anxiety). One longitudinal study [34] found no association between sedentary behaviour and risk of anxiety. One cross-sectional study found both inverse and null associations between sedentary behaviour and risk of anxiety [30] (dependent on the target group examined), whilst two cross-sectional studies [32, 36] found both positive associations and null associations (dependent on the sedentary behaviour examined). Since one strong- [31], three moderate- [33, 35, 36] and three weak-quality studies [29, 32, 37] demonstrated at least one positive relationship between sedentary behaviour and anxiety risk, based on the best-evidence synthesis, there was *moderate evidence* for the overall relationship between sedentary behaviour and anxiety risk.

### Sitting time

A total of five studies (four cross-sectional, one longitudinal) examined the association between overall sitting time and risk of anxiety [29, 33, 35–37] in adults, with all but one [29] finding a positive association between sitting time and anxiety risk. Although the longitudinal study by Uijtdewilligen and colleagues [37] demonstrated this positive association, the direction of this relationship indicated that anxiety symptoms during adolescence were predictive of sitting time in adulthood. Furthermore, Rebar et al. found that although transport-related and overall sitting time were associated with higher risk of anxiety, sitting time undertaken for work purposes or during leisure time were not associated with anxiety risk [36]. Based on the consistent findings of the three moderate- [33, 35, 36] and one weak-quality [37] studies, the best-evidence synthesis demonstrated there was *moderate evidence* for the positive relationship between sitting time and anxiety risk.

### Screen-time (i.e. combined TV, computer and/or electronic games)

A total of four studies (three cross-sectional, one longitudinal) examined the relationship between screen-time and risk of anxiety [30–32, 34]. Of those, two cross-sectional studies (one in a sample of adolescence and one in adults) showed a positive association between screen-time (i.e. combined TV and computer use) and anxiety risk [31, 32]. More specifically, Cao and colleagues [31] showed that high school students who spent more than 2-hours a day engaged in screen-based behaviours were 36 % more likely to experience anxiety symptoms than those who engaged in less than 2-hours a day. In contrast, one cross-sectional study [30] showed an inverse association between screen-time (i.e. combined TV, computer, and electronic games use) and anxiety risk amongst 5-year old girls (results were not significant for boys). Those findings suggested that girls who spent less than 2-hours in screen-based entertainment were more likely to suffer symptoms related to anxiety. However, Sanchez-Villegas et al. [34] found no association between self-reported TV/computer use time and anxiety (defined as previous or current diagnoses of anxiety from a health professional). Based on the inconsistent findings of the one strong- [31], two moderate- [30, 34] and one weak-quality [32] studies, there was *insufficient evidence* for the relationship between screen time and anxiety risk.

### Television viewing

Three cross-sectional studies amongst adults examined the relationship between television viewing and risk of anxiety [29, 32, 36]. Two of those studies showed positive associations [29, 32], suggesting that television viewing was associated with an increased likelihood of anxiety symptoms. However, based on the inconsistent findings of the one moderate- [36] and two weak-quality [29, 32] studies, there was *insufficient evidence* for the relationship between television viewing and anxiety risk.

### Computer use

Two cross-sectional studies investigated the association between computer use and risk of anxiety in adults [32, 36]. Although the moderate quality study by Rebar et al. showed a positive association between sitting at the computer and anxiety symptoms [36], the weak quality study by de Wit et al. showed no significant associations between the two factors [32]. However, that study included only a measure of computer use during leisure-time and thus it is not clear as to whether these results would remain similar for computer use undertaken for work purposes. Based on these inconsistent findings there was *insufficient evidence* for the relationship between computer use and anxiety risk.

## Discussion

This is the first review to examine evidence regarding the association between sedentary behaviour and risk of anxiety. It is important to better understand this relationship as this information may help to inform the development of lifestyle change strategies for reducing the risk of anxiety in different population groups. It is clear from this review that the current body of evidence exploring the relationship between sedentary behaviour and risk of anxiety is limited, with only nine studies currently been published. On the balance, however, most studies (78 %) found at least one positive association between sedentary behaviour and anxiety risk [29, 31–33, 35–37]. In other words, there is *moderate evidence* suggesting that engaging in overall sedentary behaviour was linked to an increased risk of anxiety. These findings are similar to those found in previous reviews that have assessed the relationship between sedentary behaviour and other specific mental health outcomes such as depression [12]. However, in our review, based on the best-evidence synthesis and when considering the different types of sedentary behaviour separately, moderate evidence was found for the positive relationship between sitting time and anxiety risk, whilst inconsistent evidence was found for the relationship between screen time, television viewing time, computer use, and anxiety risk.

There is currently limited insight into the underlying mechanisms that may explain this positive relationship between sedentary behaviour and anxiety risk. As already discussed, plausible biological pathways may include central nervous system arousal [21], sleep disturbances [22] or poor metabolic health [23] resulting from engagement in sedentary behaviour. Furthermore, drawing from previously suggested hypotheses that have been used to explain the link between sedentary behaviour and other mental disorders (i.e. depression), it could be the displacement of physical activity when engaging in sedentary behaviour that explains the relationship with increased anxiety risk, since physical activity has been shown to be beneficial in reducing anxiety in both children/adolescents [38] and adults [39]. Alternatively, the link may be explained by a social withdrawal theory which posits that engaging in prolonged sedentary behaviours, such as television viewing, may lead to social solitude and withdrawing from interpersonal relationships which has been linked to increased feelings of social anxiety [40]. On the other hand, it may be that those suffering anxiety symptoms are more inclined to engage in sedentary behaviours as a means of coping with anxiety, as has been suggested in previous research amongst adolescence with social physique anxiety [41].

In contrast, one cross-sectional study included in our review [30] showed that sedentary behaviour was inversely associated with risk of anxiety in girls (i.e. those who spent less than 2-hours in screen-based entertainment were more likely to suffer symptoms related to anxiety). These findings may suggest that screen-based entertainment could be beneficial for relieving/managing anxiety symptoms in children. Alternatively, due to the cross-sectional nature of the study, they may suggest that children with higher levels of anxiety may be less likely to engage in screen-based entertainment and perhaps more likely to engage in other non-screen based sedentary activities such as reading/studying. There is a small body of literature that suggests some forms of sedentary behaviour may in fact have a positive impact on mental health, specifically depressive symptoms [42, 43], however, further intervention and prospective studies are required to determine the direction of the relationship between sedentary behaviour and anxiety symptoms.

All studies included in this review were limited by several methodological weaknesses. For example, most studies (7/9) employed a cross-sectional study design and therefore causality and/or direction of relationships were unable to be determined. Secondly, self, or proxy-report measures of sedentary behaviour were utilised in most (n = 8) studies with such measures increasing the likelihood of recall problems and provision of socially desirable responses and thus to overcome these limitations further research involving objective measures of sedentary behaviour (e.g. accelerometers, posture monitors [i.e. activPALs]) is recommended. However, since the relationship between sedentary behaviour and anxiety risk may be dependent on the domain/type of sedentary behaviour (an aspect of sedentary behaviour that is not able to be measured using such monitors), a combination of both objective and subjective (e.g. self-report surveys) methods is warranted. This further highlights the need for the development of valid objective measures of sedentary behaviour which assess not only the dose (e.g. frequency, duration) but also the domain (e.g. leisure, work, transport), and context (TV viewing, computer use, tablet/smart phone use) in which these behaviours occur. Thirdly, only two studies [32, 36] compared different types of sedentary behaviours and their relationship with anxiety risk and therefore we were unable to clearly determine which specific sedentary behaviours may be linked to anxiety. Further, since few studies used the same method to define/assess sedentary behaviour, and every study included a different measure of anxiety symptoms, clear-cut conclusions were difficult to determine. Finally, given that anxiety, and depression are often co-morbid disorders [44], it is difficult to disentangle the relationship sedentary behaviour has with anxiety alone compared to comorbidity associations.

This review enhances the understanding of where the field is at in terms of sedentary behaviour and anxiety research. Although only limited evidence is currently available on the association between sedentary behaviour and risk of anxiety, on the balance this evidence suggests a positive association may exist between overall sedentary behaviour (e.g. sitting time specifically) and anxiety risk, whilst inconsistent evidence remains for other types of sedentary behaviours (e.g. computer use, television viewing, screen time), and their link with anxiety risk. This review further highlights the need for more high-quality longitudinal and intervention research to confirm and disentangle cross-sectional research findings.

## Additional files

Additional file 1:Table S2. Methodological quality assessment checklist for observational studies.

## References

[1] Pate RR, Stevens J, Webber LS, Dowda M, Murray DM, Young DR (2009). Age-related change in physical activity in adolescent girls. J Adolesc Health.

[2] Must A, Tybor DJ (2005). Physical activity and sedentary behavior: a review of longitudinal studies of weight and adiposity in youth. Int J Obes Relat Metab Disord.

[3] Hu FB, Li TY, Colditz GA, Willett WC, Manson JE (2003). Television watching and other sedentary behaviors in relation to risk of obesity and type 2 diabetes mellitus in women. JAMA.

[4] Katzmarzyk PT, Church TS, Craig CL, Bouchard C (2009). Sitting time and mortality from all causes, cardiovascular disease, and cancer. Med Sci Sports Exerc.

[5] Warburton DER, Nicol CW, Bredin SSD (2006). Health benefits of physical activity: the evidence. Can Med Assoc J.

[6] Spruijt-Metz D, Nguyen-Michel ST, Goran MI, Chou C, Huang TTK (2008). Reducing sedentary behavior in minority girls via a theory-based, tailored classroom media intervention. Int J Pediatr Obes.

[7] Proper KI, Singh AS, van Mechelen W, Chinapaw MJ (2011). Sedentary behaviors and health outcomes among adults: a systematic review of prospective studies. Am J Prev Med.

[8] Thorp AA, Owen N, Neuhaus M, Dunstan DW (2011). Sedentary behaviors and subsequent health outcomes in adults a systematic review of longitudinal studies, 1996–2011. Am J Prev Med.

[9] Tremblay MS, Leblanc AG, Kho ME, Saunders TJ, Larouche R, Colley RC (2011). Systematic review of sedentary behaviour and health indicators in school-aged children and youth. Int J Behav Nutr Phys Act.

[10] Costigan SA, Barnett L, Plotnikoff RC, Lubans DR (2013). The health indicators associated with screen-based sedentary behavior among adolescent girls: a systematic review. J Adolesc Health.

[11] Chinapaw MJ, Proper KI, Brug J, van Mechelen W, Singh AS (2011). Relationship between young peoples’ sedentary behaviour and biomedical health indicators: a systematic review of prospective studies. Obesity reviews: an official journal of the International Association for the Study of Obesity.

[12] Teychenne M, Ball K, Salmon J (2010). Sedentary behavior and depression among adults: a review. IntJ Behav Med.

[13] Teychenne M, Ball K, Salmon J (2010). Physical activity, sedentary behavior and depression among disadvantaged women. Health Educ Res.

[14] Nihill GFJ, Lubans DR, Plotnikoff RC (2013). Associations between sedentary behavior and self-esteem in adolescent girls from schools in low-income communities. Mental Health and Physical Activity.

[15] Australian Institute of Health and Welfare: Australia’s Health 2010. Australia’s health series. Volume 12. Australian Institute of Health and Welfare: Canberra, Australia; 2010.

[16] Australian Bureau of Statistics (2008). National Survey of Mental Health and Wellbeing.

[17] World Health Organisation (2013). Global Health Estimates summary tables.

[18] Gale C, Oakley-Browne M (2004). EBMH notebook: generalised anxiety disorder. Evid Based Ment Health.

[19] Roy-Byrne PP, Davidson KW, Kessler RC, Asmundson GJ, Goodwin RD, Kubzansky L (2008). Anxiety disorders and comorbid medical illness. Gen Hosp Psychiatry.

[20] Australian Bureau of Statistics (2010). Measures of Australia’s Progress.

[21] Wang X, Perry AC (2006). Metabolic and physiologic responses to video game play in 7- to 10-year-old boys. Arch Pediatr Adolesc Med.

[22] Dworak M, Schierl T, Bruns T, Struder HK (2007). Impact of singular excessive computer game and television exposure on sleep patterns and memory performance of school-aged children. Pediatrics.

[23] Mommersteeg PM, Herr R, Zijlstra WP, Schneider S, Pouwer F (2012). Higher levels of psychological distress are associated with a higher risk of incident diabetes during 18 year follow-up: results from the British household panel survey. BMC Public Health.

[24] Biddle SJH, Asare M (2011). Physical activity and mental health in children and adolescents: a review of reviews. Br J Sports Med.

[25] Conn VS (2010). Anxiety outcomes after physical activity interventions: meta-analysis findings. Nurs Res.

[26] De Mello MT, Lemos V, Antunes HKM, Bittencourt L, Santos-Silva R, Tufik S (2013). Relationship between physical activity and depression and anxiety symptoms: a population study. J Affect Disord.

[27] Taintor Z, Rosenthal RN (2009). Microprocessor abuse and internet addiction.

[28] National Collaborating Centre for Methods and Tools (2008). Quality Assessment Tool for Quantitative Studies.

[29] Granner ML, Mburia-Mwalili A (2010). Correlates of television viewing among african american and caucasian women. Women & Health.

[30] Griffiths LJ, Dowda M, Dezateux C, Pate R. Associations between sport and screen-entertainment with mental health problems in 5-year-old children. Int J Behav Nutr Phys Act. 2010;7. 10.1186/1479-5868-7-30 PMC2867988 20409310

[31] Cao H, Qian Q, Weng T, Yuan C, Sun Y, Wang H (2011). Screen time, physical activity and mental health among urban adolescents in China. Prev Med.

[32] de Wit L, van Straten A, Lamers F, Cuijpers P, Penninx B (2011). Are sedentary television watching and computer use behaviors associated with anxiety and depressive disorders?. Psychiatry Res.

[33] Kilpatrick M, Sanderson K, Blizzard L, Teale B, Venn A (2013). Cross-sectional associations between sitting at work and psychological distress: Reducing sitting time may benefit mental health. Mental Health and Physical Activity.

[34] Sanchez-Villegas A, Ara I, Guillen-Grima F, Bes-Rastrollo M, Varo-Cenarruzabeitia JJ, Martinez-Gonzalez MA (2008). Physical activity, sedentary index, and mental disorders in the SUN Cohort Study. Med Sci Sports Exerc.

[35] Sloan RA, Sawada SS, Daniel G, Liu Y, Biddle SJH, Blair SN (2013). Associations of sedentary behavior and physical activity with psychological distress: a cross-sectional study from Singapore. BMC Public Health.

[36] Rebar AL, Vandelanotte C, Van Uffelen J, Short C, Duncan MJ (2014). Associations of overall sitting time and sitting time in different contexts with depression, anxiety, and stress symptoms. Mental Health and Physical Activity.

[37] Uijtdewilligen L, Singh AS, Twisk JWR, Koppes LLJ, van Mechelen W, Chinapaw MJM. Adolescent predictors of objectively measured physical activity and sedentary behaviour at age 42: The Amsterdam Growth and Health Longitudinal Study (AGAHLS). Int J Behav Nutr Phys Act. 2011;8. 10.1186/1479-5868-8-107 PMC3198875 21961795

[38] Larun L, Nordheim LV, Ekeland E, Hagen KB, Heian F (2006). Exercise in prevention and treatment of anxiety and depression among children and young people. Cochrane Database Syst Rev.

[39] Strohle A (2009). Physical activity, exercise, depression and anxiety disorders. J Neural Transm.

[40] Rubin KH, Burgess KB, Vasey MW, Dadds MR (2000). The developmental psychopathology of anxiet. Social withdrawal and anxiety.

[41] Sabiston CM, Sedgwick WA, Crocker PRE, Kowalski KC, Mack DE (2007). Social physique anxiety in adolescence: an exploration of influences, coping strategies, and health behaviors. J Adolesc Res.

[42] Kraut R, Kiesler S, Boneva B, Cummings J, Helgeson V, Crawford A (2002). Internet paradox revisited. J Soc Issues.

[43] Shaw LH, Gant LM (2002). Users divided? Exploring the gender gap in Internet use. Cyberpsychol Behav: the impact of the Internet, multimedia and virtual reality on behavior and society.

[44] Gorman JM (1996). Comorbid depression and anxiety spectrum disorders. Depress Anxiety.

